# Association of antenatal corticosteroids with mortality and morbidities in very preterm infants born to women with hypertensive disorders of pregnancy: a multicenter prospective cohort study

**DOI:** 10.1186/s12884-023-06195-z

**Published:** 2024-02-05

**Authors:** Xiao-Yu Dong, Jian-Hong Qi, Qing-Cui Zhuo, Yan-Jie Ding, Xin Qiao, Yan Wang, De-Juan Yang, Dan Li, Li Li, Hai-Yan Jiang, Qiong-Yu Liu, Zhong-Liang Li, Xiang Zhang, Bing-Jin Zhang, Yong-Hui Yu

**Affiliations:** 1 Department of Pediatrics, Shandong Provincial Maternal and Child Health Care Hospital Affiliated to Qingdao University, Jinan, China; 2 Department of Neonatology, Shandong University; Shandong Provincial Hospital Affiliated to Shandong First Medical University, No. 324, Jingwu Road, HuaiYin District, Jinan, Shandong 250021 China; 3 Department of Neonatology, Qilu Hospital of Shandong University, Jinan, China; 4 Department of Neonatology, Yantai Yuhuangding Hospital, Yantai, China; 5 Department of Neonatology, Jinan Maternity and Child Healthcare Hospital, Jinan, China; 6 Department of Neonatology, The Affiliated Taian City Central Hospital of Qingdao University, Taian, China; 7 Department of Neonatology, The First Affiliated Hospital of Shandong First Medical University, Jinan, China; 8 Department of Neonatology, Liaocheng People’s Hospital, Liaocheng, China; 9 Department of Neonatology, Linyi People’s Hospital, Linyi, China; 10 Department of Pediatrics, The Third Hospital of Baogang Group, Baotou, China; 11 Department of Neonatology, Women and Children’s Healthcare Hospital of Linyi, Linyi, China; 12 Department of Neonatology, W.F. Maternal and Child Health Hospital, Weifang, China; 13 Department of Neonatology, Hebei Petro China Central Hospital, Langfang, China; 14 Department of Neonatology, Shengli Olifield Central Hospital, Yantai, China

**Keywords:** Hypertensive disorders of pregnancy, Antenatal corticosteroids, Very preterm infants, Pulmonary hemorrhage, Mortality, Morbility, Mechanical ventilation

## Abstract

**Background:**

Hypertensive disorders of pregnancy (HDP) is the most common cause of indicated preterm delivery, but the impact of prenatal steroid exposure on the outcomes of preterm infants born to HDP mothers, who may be at risk for intrauterine hypoxia-ischemia, remains uncertain. The study objective is to evaluate the mortality and morbidities in HDP for very preterm infants (VPIs) exposed to different course of ANS.

**Methods:**

This is a prospective cohort study comprising infants with < 32 weeks gestation born to women with HDP only from 1 Jan. 2019 to 31 Dec. 2021 within 40 participating neonatal intensive care units (NICUs) in Sino-northern network. ANS courses included completed, partial, repeated, and no ANS. Univariate and multivariable analyses were performed on administration of ANS and short-term outcomes before discharge.

**Results:**

Among 1917 VPIs born to women with HDP only, 987(51.4%) received a complete course of ANS within 48 h to 7 days before birth, 560(29.2%) received partial ANS within 24 h before delivery, 100(5.2%) received repeat ANS and 270 (14.1%) did not receive any ANS. Compared to infants who received complete ANS, infants unexposed to ANS was associated with higher odds of death (AOR 1.85; 95%CI 1.10, 3.14), Severe Neurological Injury (SNI) or death (AOR 1.68; 95%CI 1.29,3.80) and NEC or death (AOR 1.78; 95%CI 1.55, 2.89), the repeated ANS group exhibits a significant negative correlation with the duration of oxygen therapy days (correlation coefficient − 18.3; 95%CI-39.2, -2.1). However, there were no significant differences observed between the full course and partial course groups in terms of outcomes. We can draw similar conclusions in the non-SGA group, while the differences are not significant in the SGA group. From KM curve, it showed that the repeated group had the highest survival rate, but the statistical analysis did not indicate a significant difference.

**Conclusions:**

Even partial courses of ANS administered within 24 h before delivery proved to be protective against death and other morbidities. The differences mentioned above are more pronounced in the non-SGA group. Repeat courses demonstrate a trend toward protection, but this still needs to be confirmed by larger samples.

**Supplementary Information:**

The online version contains supplementary material available at 10.1186/s12884-023-06195-z.

## Introduction

Hypertensive disorders of pregnancy (HDP) is one of the main antecedents of preterm birth, which is the most common cause of indicated preterm delivery [1]. HDP also adversely affects various systems in very preterm infants, increasing the risk of conditions such as bronchopulmonary dysplasia (BPD), neonatal respiratory distress syndrome (NRDS), extrauterine growth retardation (EUGR), and hypoglycemia [2]. Administering antenatal corticosteroids (ANS) therapy is an effective intervention that promotes fetal lung maturation and improves metabolic transition, thereby reducing morbidity and mortality in very preterm infants [3–5].

However, in cases of HDP, particularly preeclampsia, the fetus is exposed to adverse intrauterine conditions even before ANS therapy is administered. These conditions, including oxygen deficiency, inadequate nutrition, oxidative stress, inflammatory cytokines, and anti-angiogenic factors can lead to increased uterine pressure and higher steroid levels in the fetus [6, 7]. As a result, the expected benefits of ANS treatment on neonatal outcomes may not be fully realized in pregnancies complicated by HDP. Besides, preterm infants with HDP have a high incidence of intrauterine growth restriction (IUGR), and the use of prenatal hormones for IUGR remains controversial.

The optimal course of ANS is typically recommended within 24 h to 7 days before delivery [8]. However, in the long-term management of HDP, the prediction of preterm birth has a high negative predictive value and a low positive predictive value [9]. This uncertainty often leaves clinicians perplexed about the need for repeated administration. Even within guidelines, there is a lack of consensus regarding repeated treatments. The International Society for the Study of Hypertension in Pregnancy does not recommend repeated ANS, other supportive guidelines vary from dosing interval [10]. Additionally, Effective Perinatal Intensive Care in Europe demonstrated that administering antenatal corticosteroids just a few hours prior to birth significantly reduced infant mortality with maximal reductions achieved at 18 to 24 h following administration [11]. However, there is a lack of evidence regarding the efficacy of urgent administration in the population of mothers with HDP.

Thus, the present prospective cohort study aims to investigate the effect of exposure to different ANS for mothers with HDP in very preterm infants, especially for partial ANS for rescue group and repeated group, so as to provide evidence for clinical decision-making.

## Materials and methods

### Study design and population

The Care-Preterm Study was prospective cohort study based on Sino-Neonatal Network, any relevant data relating to the pregnant mothers and their very preterm infants was collected prospectively, VPIs born at < 32 completed weeks of gestation were admitted to one of the 40 participating neonatal intensive care units (NICUs) in our network. The database provided maternal, delivery and neonatal data up until the first NICU discharge. All data was collected by trained staff using a standardised operating procedure [12].

The study population included very preterm infants who were admitted to the NICUs of 40 level-III hospitals in SNN. Only infants delivered by indicated preterm delivery due to HDP were included, mothers existed with diseases other than HDP and those existed with other indications for preterm delivery were excluded. Infants who underwent withdrawal of treatment due to socio-economic factors or infants with a congenital anomaly or with unknown timing or times of ANS were alsobexcluded. The study protocol was approved by each center’s institutional review board.

### Definitions

Hypertensive disorders of pregnancy comprised chronic or pregnancy-induced hypertension, which is defined as a systolic blood pressure above 140 mmHg or a diastolic blood pressure above 90 mmHg recorded before or during the current pregnancy, with or without edema and proteinuria. HDP includes gestational hypertension, pre-eclampsia, eclampsia, as well as pregnancy complicated by chronic hypertension and chronic hypertension with superimposed pre-eclampsia [13].

Pregnant women with pre-eclampsia who are expected to deliver within one week and are less than 34 weeks pregnant should receive glucocorticoid therapy. Mothers were considered to have received a complete course if they had received 5 mg or 6 mg intramuscular injections of dexamethasone every 12 h for a total of 4 doses or 12 mg intramuscular injections of betamethasone once daily for 2 days. A partial course of antenatal corticosteroid was defined as dexamethasone administration at a dosage of 6 mg intramuscularly every 12 h within 24 h before delivery. For cases where a complete course has been given seven days prior but a repeat course has not been completed within seven days of a rescue course, they will also be classified under the partial course group. Mothers who were considered to have received a repeat course were those women at risk for very preterm birth, who had received a course of corticosteroids ≥ 7 days prior, and who were prescribed repeat administrations of intramuscular dexamethasone if the risk of very preterm birth remained. Furthermore, we categorized the group that received ANS 1 week prior to delivery and did not receive any further doses as the no ANS group.

The primary outcome measure was death before hospital discharge. Secondary outcome measures included: NRDS (oxygen requirement, clinical diagnosis, and consistent chest radiograph), bronchopulmonary dysplasia, severe IVH (grades III or IV), periventricular leukomalacia, blood culture-proven early onset sepsis, necrotizing enterocolitis, pneumothorax, neonatal hyperglycemia, hypoglycemia, EUGR, and weight, length and head circumference at discharge.

The diagnostic criteria for clinical chorioamnionitis are as follows: maternal body temperature elevation (≥ 37.8 ℃) accompanied by 2 or more of the following abnormalities: increased heart rate (≥ 100 beats/min), increased fetal heart rate (≥ 160 beats/min), uterine tenderness, foul-smelling vaginal discharge, elevated peripheral blood white cell count (≥ 15 × 10^9/L), or a left shift in the differential count [14]. Resuscitation involves extensive measures like positive pressure ventilation, intubation, and chest compressions. Small for gestational age (SGA) is defined as birth weight below the 10th percentile of average birth weight for the same gestational age [15]. Hyperglycemia defined asblood glucose level ≥ 150 mg/dL or 8.3 mmol/L. Hypoglycemia was defined as < 40 mg/ dL in the first 24 h of life. Based on the 2013 Fenton growth curves, infants are defined as having Extrauterine Growth Restriction (EUGR) if their body weight, at discharge or corrected gestational age of 36 weeks, falls below the 10th percentile (P10) for the corresponding gestational age group [16].

NRDS severity was graded as follows [17]: Grade I: fine miliary ground-glass shadows and opacification of both lungs. Grade II: empty bronchial shadow beyond heart shadow in addition to miliary shadows. Grade III: blurred cardiac and septal margins in addition to the above shadows, and aerated bronchus sign. Grade IV: extensive white shadow (white lung), and severer aerated bronchus sign. IVH was graded in accordance with data from Papile et al. [18] and periventricular leukomalacia (PVL) was defined as proposed by de Vries et al [19]. Severe Neurological Injury (SNI) includes severe IVH (grades III or IV) and any level of periventricular leukomalacia. Retinopathy of prematurity (ROP) was defined in accordance with the International Classification for Retinopathy of Prematurity [20] and NEC according to Bell et al., where stage two or higher was considered as diagnostic [21]. Severe bronchopulmonary dysplasia (BPD) was defined as the need for ≥ 30% oxygen at a postmenstrual age of 36 weeks [22]. The denominator for ROP includes infants who underwent retinopathy of prematurity screening, while the denominator for BPD comprises infants who survived beyond 28 days.

### Statistical analysis

The data is presented as numbers and proportions unless stated otherwise. Differences between treatment groups in terms of baseline maternal characteristics and maternal delivery characteristics were determined using a 2-tailed or χ2 test and Fisher exact test for dichotomous variables and a Wilcoxon rank sum test for continuous or ordinal variables. The association between different ANS exposures and outcomes was analysed using a multiple logistic regression model adjusting for the factors with *p* < 0.1 in the univariate analysis. We also corrected for center effect by encoding multiple centers as dummy variables and including these dummy variables as independent variables in the logistic regression model. Linear regression was used to determine outcomes of growth (height, weight, and head circumference at discharge) and ventilation days, oxygen therapy days. After ensuring that there is no collinearity among variables through statistical hypothesis testing, we can proceed to use the Cox proportional hazards regression analysis to assess cumulative infant survival. Results are presented as odds compared with the reference category of a complete course before birth, odds ratio (OR) = 1.00. All tests were two-sided, and a *P*-value of less than 0.05 was considered statistically significant. All statistical analyses were conducted using SPSS v.25.0 (SPSS Inc., Chicago, Illinois).

## Results

### Study participants

**Fig. 1:**
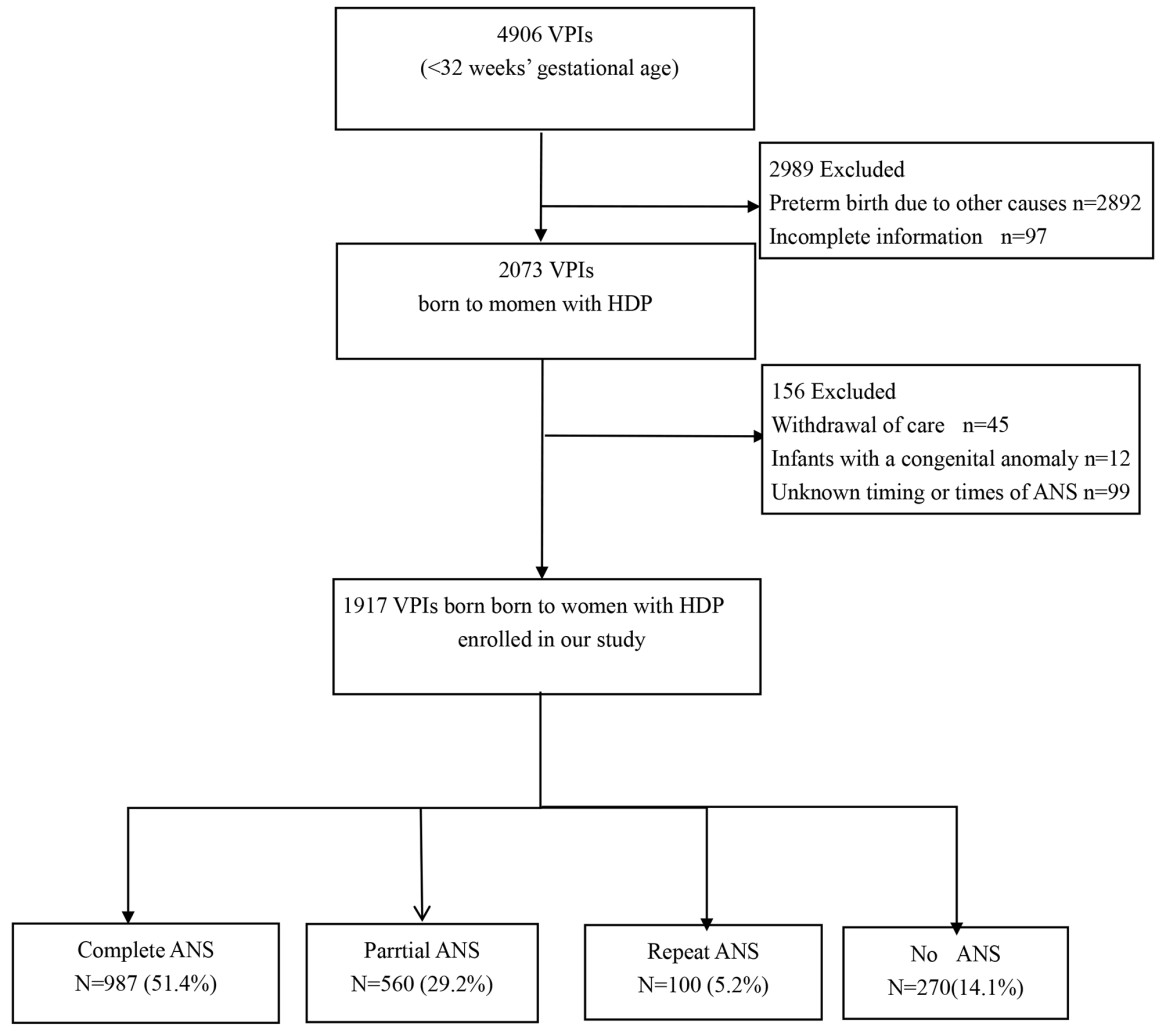
Flowchart for inclusion. HDP- hypertensive disorders of pregnancyis, ANS- Antenatal corticosteroids

A total of 4906 infants with GA less than 32 were born at SNN centers. Preterm delivery induced by other reasons and with incomplete information were excluded, leaving 2073 infants whose mothers presented with HDP to be included. We then excluded those who were given withdrawal of care for socioeconomic concerns, combined with severe malformations, and unknown times or timing of ANS, leaving a total of 1917 infants to be enrolled in this study. (Fig. 1). Among them, 987(51.4%) received a complete course of ANS within 48 h to 7 days before birth,560(29.2%) received incomplete courses within 24 h from the 1st dose,100(5.2%), who had received a course of corticosteroids ≥ 7 days previously, were prescribed repeat courses, the maximum number of courses was two, a total of 1647(85.9%) had been exposed to ANS, whereas 270(14.1%) were unexposed to ANS.

### Baseline characteristics

Maternal and infant characteristics for the different courses of ANS are described in Table 1. Infants exposed to complete ANS were more likely to delivery by cesarean section (*P* < 0.001) and were more likely conceived through assisted reproductive technology (ART). Differences were observed among the treatment groups for hypertension classification (*P* = 0.006). The chronic hypertension group is more likely to receive the complete course of ANS. Pre-eclampsia is more likely to be treated with the complete or partial course, higher proportion of eclampsia and HELLP syndrome is observed in the group that did not receive ANS. There are discernible variations in birth weight among the different groups, with the no ANS group exhibiting the highest birth weight and the complete group displaying the lowest birth weight. The pattern is consistent with the birth weight Z-scores as well. ANS is administered to a higher proportion of newborns inborn. In the no ANS group, more infants had Apgar scores less than 7 at 5 min (*p* < 0.001).

**Table 1:** Maternal and peripartum characteristics between 4 groups

	Complete ANS	Partial ANS	Repeat ANS	No ANS	*P*
	N = 987	N = 560	N = 100	N = 270	
Maternal age, year	33(30,37)	33(29,37)	33(29,37)	33(30,37)	0.713
Assisted reproductive technology	102(10.3)	47(8.4)	7(7.0)	14(5.2)	0.052
Cesarean section	955(96.8)	522(93.2)	93(93.0)	235(87.0)	<0.001
Classification of hypertension					0.006
Chronic hypertension	90(9.1)	61(10.9)	10(10.0)	24(8.9)	
Gestational hypertension	166(16.8)	136(24.3)	28(28.0)	66(24.4)	
Preeclampsia	665(67.4)	315(56.3)	54(54.0)	160(59.3)	
Eclampsia	31(3.1)	21(3.8)	3(3.0)	10(3.7)	
HELLP syndrome	35(3.5)	27(4.8)	5(5.0)	10(3.7)	
PPROM	88(8.9)	71(12.7)	9(9.0)	2(8.1)	0.074
Chorioamnionitis	24(2.4)	15(2.7)	3(3.0)	1(0.4)	0.156
Gestational Age, wk	30(29,31)	30(29,31)	30(29,31)	30(29,31)	0.463
Birth weight (g), mean (SD)	1190(1000,1370)	1200(1000,1423)	1245(1100,1450)	1250(1050,1430)	0.007
Birth weight z-scores	−0.105(−0.549,0.404)	−0.004(−0.546,0.537)	0.144(−0.421,0.539)	0.005(−0.9,0.677)	0.026
Birth head circumference, cm	27(25.5,28)	27(25,28.5)	27(26,29)	27(26,29)	0.434
Male gender	496(50.3)	30(53.8)	43(43.0)	141(52.2)	0.202
Multiples	145(14.7)	74(13.2)	15(15.0)	23(8.5)	0.066
Inborn	974(98.7)	548(97.9)	100(100.0)	243(90.0)	<0.001
Apgar <7 at 5 min	98(9.9)	81(14.5)	10(10.0)	51(18.9)	<0.001
SNAPPE-II	13(5,27)	14(5,30)	13(0,27.8)	14(5,28)	0.167

Abbreviation: ACS, antenatal corticosteroid; HELLP syndrome, Hemolysis, Elevated Liver enzymes, Low Platelet count syndrome; PPROM, Preterm Prelabor Rupture of Membranes; SNAPPE-II, Score for Neonatal Acute Physiology with Perinatal Extension-II

### Adverse neonatal outcomes

The clinical outcomes during hospital stay are described in Table 2. When comparing the outcomes of death, SNI or death, and NEC or death, the occurrence rate of repeat ANS is the lowest, while the no ANS group has the highest rate. The repeat ANS group also has the shortest duration of oxygen use. After adjustment for potential confounder, compared to infants who received complete ANS, infants unexposed to ANS was associated with higher odds of death (AOR 1.85; 95%CI 1.10, 3.14), SNI or death (AOR 1.68; 95%CI 1.29,3.80) and NEC or death (AOR 1.78; 95%CI 1.55, 2.89). (Table 3). In comparison to the complete ANS group, the repeated ANS group exhibits a significant negative correlation with the duration of oxygen therapy days (correlation coefficient − 18.3; 95%CI-39.2, -2.1) (Table 4). There were no differences observed among the three groups in terms of weight, head circumference, and body length at discharge.

**Table 2:** Outcomes of infants exposed to different courses of ANS

	Complete ANS	Partial ANS	Repeat ANS	No ANS	*P*
	N = 987	N = 560	N = 100	N = 270	
Resuscitation	602(61.0)	350(62.5)	58(58)	167(61.9)	0.834
Intubation in DR	248(25.1)	164(29.3)	26(26.0)	87(32.2)	0.078
PS	398(40.3)	245(43.8)	31(31)	107(39.6)	0.101
PS ≥ 2 times	106(10.7)	71(12.7)	10(10.0)	27(10)	0.577
Hyperglycemia	77(7.8)	49(8.8)	5(5.0)	15(5.6)	0.299
Hypoglycemia	151(15.3)	67(12.0)	15(15.0)	45(16.7)	0.216
Ventilation days/length of stay	0(0,115)	0(0,128)	0(0,82)	19(0,125)	0.274
Oxygen therapy days/length of stay	347(126,706)	386(139,717)	263(29,540)	304(104,639)	0.043
Pneumothorax	8(1.1)	4(1.0)	2(3.3)	5(2.6)	0.231
Pulmonary hemorrhage	46(14.7)	35(6.3)	8(8.0)	15(5.6)	0.366
NRDS (3–4)	206(20.9)	103(18.4)	18(18)	45(18.0)	0.370
BPD^a^	95/827(11.5)	47/455(10.3)	14/85(16.5)	21/202(10.4)	0.409
Death or BPD	75(14.7)	75(13.4)	16(16.0)	44(16.3)	0.694
SNI	15(1.5)	13(2.3)	0(0)	5(1.9)	0.361
Death or SNI	64(6.5)	39(7.0)	2(2.0)	27(10.0)	0.045
NEC	21(2.1)	12(2.1)	2(2.0)	9(3.3)	0.678
Death or NEC	73(7.4)	38(6.8)	3(3.0)	30(11.1)	0.038
ROP^b^	42(6.1)	14(3.4)	5(6.5)	7(3.9)	0.178
Death or ROP	95(9.6)	43(7.7)	7(7.0)	31(11.5)	0.258
EUGR	424(46.2)	266(51.8)	48(49.5)	99(41.8)	0.054
Head circumference at discharge	31(30,32)	31(30,32)	31(30,32)	31(30,32.5)	0.103
Length at discharge	44(43,46)	44(42.5,46)	44(43,45)	44.5(42,46)	0.461
Weight at discharge	2100(1970,2780)	2077(1930,2257.5)	2100(1960,2300)	2100(1955,2340)	0.174
Death	53(5.4)	29(5.2)	2(2.0)	24(8.9)	0.042

Abbreviations: DR, delivery room; PS, Pulmonary Surfactant; ANS, Antenatal corticosteroids; NRDS, neonatal respiratory distress syndrome;EOS, early-onset sepsis; BPD, bronchopulmonary dysplasia; IVH, intraventricular hemorrhage; NEC, necrotizing enterocolitis; ROP, retinopathy of prematurity

^a^BPD among survivors at 28 days

^b^ROP among infants conducted eye fundus examination

**Table 3:** Multivariable logistic analysis of association between outcomes and different exposure of ANS

	Complete ANS	Partial ANS	Repeat ANS	No ANS
	N = 987	N = 560	N = 100	N = 270
		AOR^c^(95%CI)	AOR (95%CI)	AOR (95%CI)
Resuscitation	1	1.31(0.90,1.41)	0.91(0.58,1.40)	1.19(0.76,1.78)
Intubation in DR	1	1.35(0.92,1.86)	1.35(0.82,2.25)	1.38(0.90,1.82)
PS	1	1.09(0.87,1.36)	0.80(0.50,1.27)	1.25(0.93,1.42)
PS ≥ 2 times	1	1.26(0.91,1.76)	1.05(0.52,2.12)	1.01(0.63,1.61)
Pneumothorax	1	0.97(0.59,4.23)	3.86(0.92,19.4)	2.79(0.80,9.74)
Pulmonary hemorrhage	1	1.58(0.94,2.83)	2.76(0.98,5.59)	1.53(0.76,2.82)
3–4 NRDS	1	0.78(0.55,1.32)	0.78(0.62,1,1.83)	0.85(0.63,1.62)
BPD^a^	1	0.88(0.69,1.38)	1.85(0.99,3.77)	0.92(0.83,1.79)
Death or BPD	1	0.97(0.03,1.29)	1.46(0.79,2.94)	1.38(0.89,186)
SNI	1	1.70(0.79,3.68)	-	1.56(0.54,3.72)
Death or SNI	1	1.33(0.89,1.82)	0.48(0.18,1.35)	1.68(1.29,3.80)
NEC	1	1.45(0.63,3.26)	0.97(0.43,2.00)	1.31(0.45,4.65)
Death or NEC	1	0.89(0.62,1.46)	0.56(0.29,1.75)	1.78(1.55,2.89)
ROP^b^	1	0.58(0.31,1.08)	1.07(0.42,2.87)	0.68(0.43,1.79)
Death or ROP	1	0.90(0.68,1.14)	0.78(0.36,1.82)	1.51(0.80,1.92)
EUGR	1	1.56(0.99,2.02)	1.46(0.65,2.82)	0.92(0.67,1.25)
Death	1	0.93(0.57,1.50)	0.39(0.1,1.68)	1.85(1.10,3.14)

Abbreviations: ANS, Antenatal corticosteroids; NRDS, neonatal respiratory distress syndrome; BPD, bronchopulmonary dysplasia; IVH, intraventricular hemorrhage; NEC, necrotizing enterocolitis; ROP, retinopathy of prematurity

^a^BPD among survivors at 28 days

^b^ROP among infants conducted eye fundus examination

^c^Adjusted for assisted reproductive technology, cesarean section, PROM, birth weight z-scores, multiples, inborn and Apgar <7 at 5 min

**Table 4:** Linear Regression analysis of association between outcomes and different exposure of ANS

	Complete ANS N = 987	Partial ANS N = 560	Repeat ANS N = 100	No ANS N = 270
		estimated changes (95% CI)^a^	estimated changes (95% CI)^a^	estimated changes (95% CI)^a^
Oxygen therapy days/ length of stay	1	9.1(-12.1, 32.5)	-18.3(-39.2, -2.1)	-8.8(-37.0,22.1)
Ventilation days/ length of stay	1	9.3(-9.3, 29.2)	-63.5(-129.2,,9.3)	-39.0(-88.6, 8.9)
Head circumference at discharge	1	-0.52(-0.81, 0.44)	-0.23(-0.76, 0.51)	-0.33(-0.48, 0.30)
Length at discharge	1	-0.35(-0.75, 0.08)	-0.38(-1.6, 0.52)	-0.33(-0.89, 0.45)
Weight at discharge	1	-45.9(-83.9, 7.4)	8.77(-69.5, 88.0)	8.72(-43.0, 67.1)

Abbreviations: ANS- Antenatal corticosteroids

^a^Adjusted for assisted reproductive technology, cesarean section, PROM, birth weight z-scores, multiples, inborn and Apgar <7 at 5 min

**Fig. 2:**
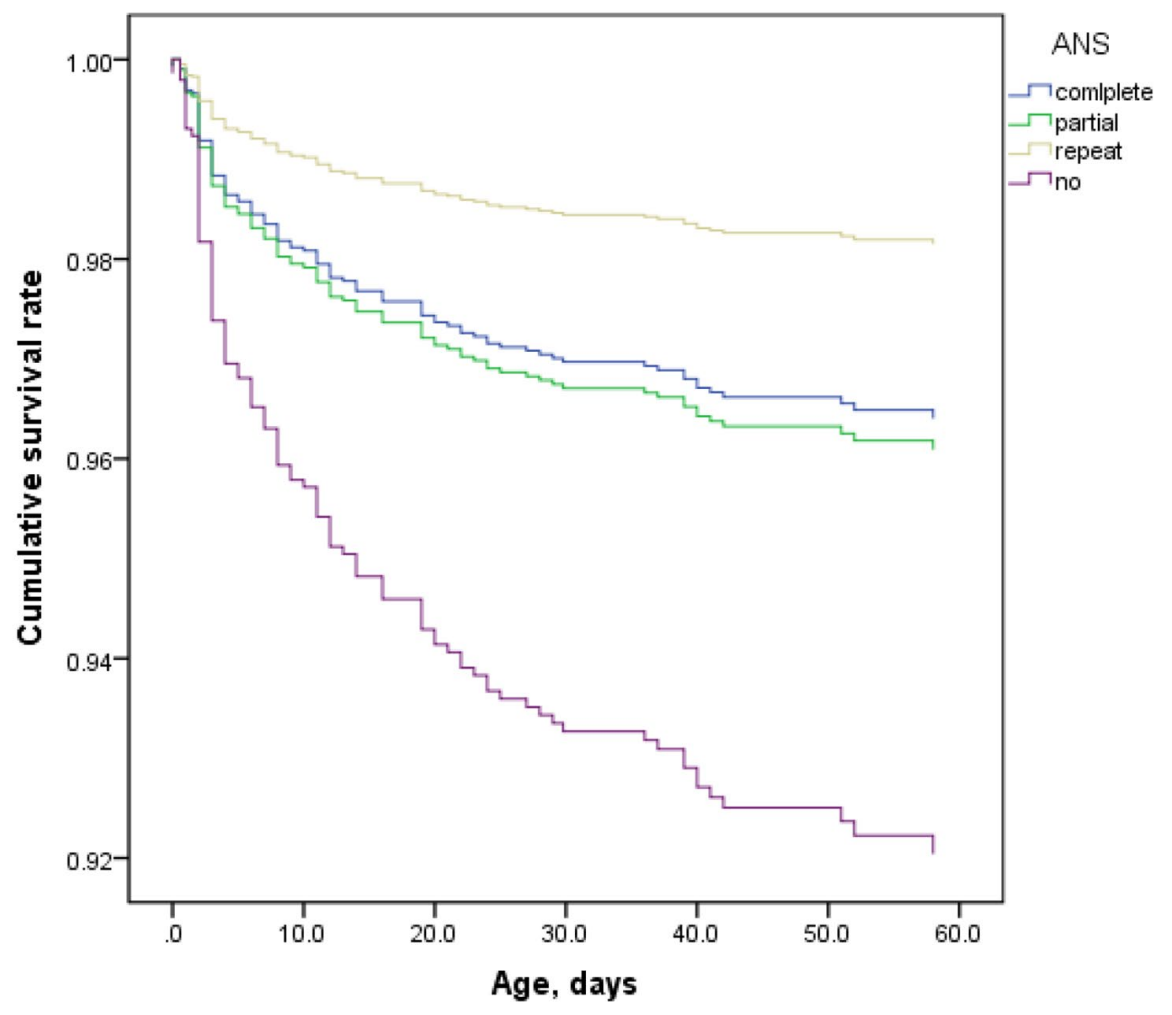
Survival curve adjusted for covariates for very preterm infants according to antenatal corticosteroid (ACS) treatment and courses

Kaplan-Meier curves Kaplan-Meier curves for duration up to 60 days showed that there were significant difference between no ANS group and complete group (AOR 2.16; 95%CI 1.41,3.68) (Fig. 2) In the graph, it showed that the repeated group had the highest survival rate, but the statistical analysis did not indicate a significant difference (AOR 0.48; 95%CI 0.15,2.03). We defined survival time as 100 days, and obtained similar results. (AOR 0.506; 95%CI 0.123,2.083) (Supplement Figure S1).

We separately analyzed the SGA and non-SGA groups and found that the effects of prenatal steroids are more significant in the non-SGA group. In the SGA group, we only found that a partial course of ANS is a protective factor against death or BPD (AOR 0.52; 95% CI 0.37,0.98) (Supplement Table S1). However, in the non-SGA group, the absence of ANS use increased the risk of death by 2-fold and also led to an increase in endotracheal intubation in the delivery room (AOR 1.71; 95% CI 1.33, 3.89), death or SNI (AOR 1.90; 95%CI1.22,3.83). A partial course of ANS was associated with an increased risk of endotracheal intubation in the delivery room (AOR 1.53; 95%CI 1.24,2.04) and postnatal surfactant usage≥ 2 times (AOR 1.54; 95% CI 1.19,2.57) (Supplement Table S2). In both groups, repeated courses of prenatal steroids did not result in lower discharge weight.

## Discussion

### Main findings

This multicenter cohort study showed that only half of VPIs born to women with HDP received complete ANS within 48 h to 7 days before birth. Approximately one fifth infants had no history of ANS exposure, one forth infants received partial ANS and less than 10% received repeat ANS. Compared to the complete course group, the non-use group had higher rates of mortality, mortality or NEC, and mortality or SNI, which was more pronounced in the non-SGA group. The repeated group had significantly fewer days of oxygen therapy, indicating statistical significance. Additionally, although not statistically significant, the repeated treatment group had the lowest incidence of complications and mortality, which also shows a protective trend in terms of survival rate from Kaplan-Meier curves.

### Interpretation

It is well known that ANS is one of the most effective measures in reducing mortality in preterm infants by accelerating the development of type 1 and type 2 pneumocytes and modifying alveolar structure vascularization, inducing surfactant production [23]. In our study, about one fifth infants had no history of ANS exposure, the occurrence of death and other severe morbidities increased significantly compared with ANS exposure within 1-7d before birth. This is similar to research on non-HDP cases, where the use of antenatal corticosteroids has been associated with an increased risk of necrotizing enterocolitis (NEC), intraventricular hemorrhage (IVH), and mortality [24, 25]. In our early research, we found that families of infants born before 28 weeks may not have had a very proactive attitude towards resuscitation, and may have only decided to aggressively treat their child after birth. Therefore, prenatal management may not have been sufficient, which may have been influenced by some non-medical factors [26]. Especially in primary healthcare settings, efforts should be made to increase the use of antenatal steroids, both during the process of emergency intrauterine transfer to tertiary hospitals and in communication with the pregnant woman and her family to enhance compliance. When we compare the percentage of non-utilized ANS between units, the range can be from 0 to 46%, excluding units with less than 20 cases, the range was from 5.1 to 34.8%, which indicates that there is still a lot of opportunity for quality improvement.

In cases where there is an emergency delivery due to conditions such as umbilical artery blood flow disappearance, eclampsia, and preeclampsia, our study has shown that a partial course of antenatal corticosteroids (ANS) can be effective. Reliable data from the EPICE Cohort suggests that neonatal benefits begin within a few hours of ANS administration [11]. Similarly, in our study, infants born to mothers with HDP who did not receive ANS had twice the risk of death compared to those who received partial courses of ANS. Moreover, even with a reduced dosage of ANS, there was no increase in the risk of mortality and complications compared to the standard treatment group. This raises the question of whether the increased intrauterine stress response associated with maternal hypertensive disorders of pregnancy contributes to these outcomes, which deserves further investigation. A long-term follow-up study indicates that children born extremely preterm who were exposed to a single course of ANS had a significantly lower risk of neurodevelopmental impairment [27]. However, there is still insufficient long-term follow-up data for premature infants born to mothers with HDP.

There is controversy in different medical literature relating to repeat courses, especially their security and effectiveness [28]. In our study, compared with the other groups, the repeat group has the lowest mortality rate and the minimum number of oxygen therapy days. Previous literature reported that repeat corticosteroids used ≥ 7 days after an initial course not only reduced serious neonatal morbidity and respiratory disease, but also reduced the mean birthweight and placenta weight [29]. However, in our study, repeated administration of steroids did not lead to a decrease in birth weight. Although there were some differences in birth weight among different groups, there were no significant differences in weight, head circumference, or length at discharge. Increasing number of related follow-up studies indicates that repeat ANS had no adverse effects on cardiometabolic function or other harm in mid-childhood, even in the presence of fetal growth restriction [30, 31]. The number of repeat treatment courses limited to a maximum of three have the best benefit to risk ratio [32]. More research is required to determine the potential effects of repeat ANS to short and long term outcomes.

Compared to preterm births caused by other factors, pregnancies affected by HDP have a higher incidence of IUGR [33]. The use of ANS for IUGR remains a topic of controversy. Some viewpoints suggest that fetal growth restriction is linked to placental insufficiency, which results in chronic malnutrition and hypoxia. The intrauterine stress may trigger the production of cortisol by the adrenal gland, which can increase the production of cortisol to adapt to the compromised intrauterine environment [34]. In our study, it appeared that the impact of antenatal steroids on the non-SGA group was more significant compared to the SGA group, which may be related to the intrauterine hormone exposure in the SGA group. However, it is important to note that our SGA sample size was small, particularly after grouping for ANS analysis, so caution is needed when drawing conclusions. Further research with larger sample sizes is still needed to investigate this further.

### Strengths and limitations

This was a large, multicenter cohort study, which was designed prospectively, including robust data on mothers, neonates, the ANS courses, we implemented strict quality control measures in defining and collecting the data for our study. In addition, our study included variables such as duration of medication and classification of HDP, which provided a more detailed analysis of the impact of ANS on premature birth outcomes within the HDP population. Besides, we compared the SGA and non-SGA groups within the larger HDP cohort, which to some extent enhanced the comparability of the effects of prenatal steroids on SGA. By limiting our study population to the HDP group with similar backgrounds, we were able to better assess the efficacy of prenatal steroids on SGA. Furthermore, we placed particular emphasis on repeated and partial ANS administration, which are crucial practical considerations in the management of HDP.

However, our study does have some limitations. Restricted by the amount of data, especially the repeated group and SGA group, there was no statistical significance, further study with a large sample size is warranted. Besides, there are differences in the prenatal management of different centers, we have not yet analyzed each level of center. Apart from that, the gestational age range of enrolled infants is large, we have not conducted a more detailed analysis of premature infants in different gestational age levels.

## Conclusions

In this prospective multicenter cohort study for VPIs born to maternal HDP, about one fifth infants still did not receive ANS, but even partial courses of ANS administered within 24 h before delivery proved to be protective against death and other morbidities. The differences mentioned above are more pronounced in the non-SGA group. Repeat courses demonstrate a trend toward protection, but this still needs to be confirmed by larger samples.

### Electronic supplementary material

Below is the link to the electronic supplementary material.

Supplementary Material 1

## Data Availability

The data that support the findings of this study are available from Sino-Neonatal Network, but restrictions apply to the availability of these data, which were used under license for the current study, and so are not publicly available. Data are however available from the authors upon reasonable request and with permission of Yonghui Yu - the PI of Sino-Neonatal Network.
