# Supplementary material for: Association of antenatal corticosteroids with mortality and morbidities in very preterm infants born to women with hypertensive disorders of pregnancy: a multicenter prospective cohort study
Source: BMC Pregnancy Childbirth. 2024 Feb 5;24:109. doi: 10.1186/s12884-023-06195-z (PMC10840159; doi:10.1186/s12884-023-06195-z)
Supplement: Supplementary file 1 — Supplementary Material 1 [file 12884_2023_6195_MOESM1_ESM.docx]

**SUPPLEMENT**


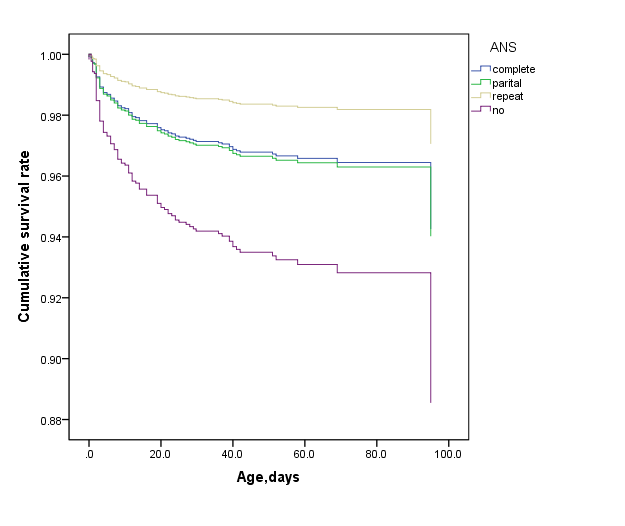


**Fig. S1** Survival curve adjusted for covariates for very preterm infants according to antenatal corticosteroid (ACS) treatment and courses.

**Table S1 a** Multivariable logistic analysis of association between outcomes and different exposure of ANS in SGA group

|  | Complete ANS | Partial ANS | Repeat ANS | No ANS |
| --- | --- | --- | --- | --- |
|  | N=183 | N=114 | N=13 | N=43 |
|  | AOR^c^(95%CI) | AOR^c^(95%CI) | AOR^c^(95%CI) | AOR^c^(95%CI) |
| Resuscitation | 1 | 1.19(0.92,2.04) | 0.34(0.12,1.34) | 0.92(0.56,1.91) |
| Intubation in DR | 1 | 1.23(0.78,2.08) | 0.69(0.27,3.22) | 1.48(0.69,3.17) |
| PS | 1 | 1.44(0.83,2.79) | 0.84(0.23,,2.19) | 1.16(0.68,2.99) |
| PS≥2 times | 1 | 0.81(0.47,2.09) | 1.39(0.24,11.73) | 0.82(0.18,2.61) |
| Hyperglycemia | 1 | 0.25(0.73,2.63) | - | 0.51(0.19,2.02) |
| Hypoglycemia | 1 | 0.72(0.43,1.61) | 0.86(0.22,3.93) | 1.43(0.71,2.95) |
| Pulmonary hemorrhage | 1 | 1.93(0.88,4.97) | 2.06(0.45,19.36) | 1.57(0.29,5.35) |
| 3-4 NRDS | 1 | 0.76(0.63,1.81) | 0.62(0.17,5.83) | 0.62(0.13,1.79) |
| BPDa | 1 | 0.66(0.40,1.98) | 1.37(0.74,.6.95) | 0.91(0.52,2.98) |
| Death or BPD |  | 0.52(0.37,0.98) | 0.99(0.39,5.77) | 1.93(0.71,3.97) |
| SNI | 1 | 1.61(0.27,10.84) | - | 2.0(0.38,23.99) |
| Death or SNI |  | 0.77(0.35,1.93) | - | 2.05(0.92,5.02) |
| NEC | 1 | 0.96(0.28,4.96) | - | 3.82(0.95,19.72) |
| Death or NEC |  | 0.87(0.36,1.88) | - | 2.01(0.60,4.97) |
| ROP^b^ | 1 | 0.35(0.19,1.32) | 2.91(0.29,24.90) | - |
| Death or ROP |  | 0.57(0.36,1.82) | 1.03(0.25,9.70) | 1.23(0.59,3.11) |
| EUGR | 1 | 1.27(0.92,2.03) | 1.55(0.47,5.88) | 1.79(0.81,4.92) |
| Death | 1 | 0.77(0.39,1.91) | - | 2.39(0.88,7.33) |
| **Table S1 b Linear Regression analysis of association between outcomes and different exposure of ANS in SGA group** | | | | |
|  | Complete ANS | Partial ANS | Repeat ANS | No ANS |
|  | N=183 | N=114 | N=13 | N=43 |
|  | estimated changes ( 95% CI)^c^ | estimated changes ( 95% CI)^c^ | estimated changes ( 95% CI)^c^ | estimated changes ( 95% CI)^c^ |
| Ventilation days/length of stay | 1 | -14.6(-84.1,53.0) | -63.0(-234.4,96.1) | -17.9(-122.1,89.0) |
| Oxygen therapy days/length of stay | 1 | -58.1(-130.9,49.2) | -286.4(-402.2,8.91) | -92.0(-232.2,79.0) |
| Head circumference at discharge | 1 | -0.061(-0.87,099) | 0.57(-1.50,2.91) | 0.23(-1.9,2.7) |
| Length at discharge | 1 | 0.12(-0.94,1.31) | 0.68(-1.79,2.90) | -1.02(-2.78,0.88) |
| Weight at discharge | 1 | -31.1(-123.2,69.3) | 142.1(-78.0,343.1) | -37.5(-179.6,97.4） |

Abbreviations: ANS, Antenatal corticosteroids; NRDS, neonatal respiratory distress syndrome; BPD, bronchopulmonary dysplasia; IVH, intraventricular hemorrhage; NEC,necrotizing enterocolitis; ROP, retinopathy of prematurity.

^a^BPD among survivors at 28 days.

^b^ROP among infants conducted eye fundus examination

^c^Adjusted for assisted reproductive technology,cesarean section,PROM, birth weight z-scores, multiples, inborn and Apgar ＜7 at 5 min

| **Table S2 a** Multivariable logistic analysis of association between outcomes and different exposure of ANS in non-SGA group | | | | |
| --- | --- | --- | --- | --- |
|  | Complete ANS | Partial ANS | Repeat ANS | No ANS |
|  | N=804 | N=446 | N=87 | N=227 |
|  | AOR^c^(95%CI) | AOR^c^(95%CI) | AOR^c^(95%CI) | AOR^c^(95%CI) |
| Resuscitation | 1 | 1.32(0.98,1.78) | 1.28(0.89,2.56) | 1.41(1.51,2.39) |
| Intubation in DR | 1 | 1.53(1.24,2.04) | 1.57(0.97,3.60) | 1.71(1.33,3.89) |
| PS | 1 | 1.75(0.96,1.84) | 0.87(0.41,1.50) | 1.01(0.72,1.50) |
| PS≥2 times | 1 | 1.54(1.19,2.57) | 1.20(0.60,2.62) | 1.35(0.89,2.03) |
| Hyperglycemia | 1 | 1.42(0.80,2.34) | 0.92(0.48,2.32) | 0.93(0.62,1.95) |
| Hypoglycemia | 1 | 0.87(0.73,1.53) | 1.47(0.69,2.50) | 1.39(0.96,2.03) |
| Pneumothorax | 1 | 0.99(0.41,3.86) | 3.23(0.66,18.90) | 1.60(0.58,6.37) |
| Pulmonary hemorrhage | 1 | 1.55(0.83,2.82) | 2.92(0.93,,6.01) | 1.81(0.88,,3.39） |
| 3-4 NRDS | 1 | 0.96(0.83,1.54) | 0.99(0.59,1.82) | 0.95(0.52,1.76) |
| BPD^a^ | 1 | 1.25(0.89,1.89) | 1.88(0.95,3.94) | 0.99(0.59,2.01) |
| Death or BPD | 1 | 1.32(0.92,1.80) | 1.72(0.88,,3.23) | 1.45(0.94,2.35) |
| SNI | 1 | 1.86(0.81,4.32) | - | 1.22(0.45,3.98) |
| Death or SNI | 1 | 1.58(0.96,2.67) | 0.63(0.29,2.84) | 1.90(1.22,3.83) |
| NEC | 1 | 1.33(0.60,2.92) | 1.13(0.44,5.26) | 1.45(0.67,3.90) |
| Death or NEC | 1 | 1.35(0.82,2.45) | 0.61(0.19,2.03) | 1.77(0.99,3.23) |
| ROP^b^ | 1 | 0.81(0.45,1.52) | 1.21(0.39,3.52) | 0.72(0.34,1.89) |
| Death or ROP | 1 | 1.22(0.69,1.70) | 0.97(0.48,2.96) | 1.44(0.89,2.92) |
| EUGR | 1 | 1.41(1.20,1.83) | 1.33(0.92,2.37) | 0.92(0.65,1.59) |
| Death | 1 | 1.42(0.85,2.56) | 0.69(0.44,2.96) | 2.22(1.09,4.77) |
| **Table S2 b** Linear Regression analysis of association between outcomes and different exposure of ANS in non-SGA group | | | | |
|  | Complete ANS | Partial ANS | Repeat ANS | No ANS |
|  | N=804 | N=446 | N=87 | N=227 |
|  | estimated changes ( 95% CI)^c^ | estimated changes  ( 95% CI)^c^ | estimated changes ( 95% CI)^c^ | estimated changes ( 95% CI)^c^ |
| Ventilation days/length of stay*1 | 1 | 9.66(-12.8,33.9) | -19.8(-64.2,23.3) | 14.9(-15.6,47.1) |
| Oxygen therapy days/length of stay | 1 | 28.3(-9.8,66.9） | -38.9(-107.1,35.3) | -25.9(-78.9,26.7) |
| Head circumference at discharge | 1 | -0.69(-0.90,-0.36) | -0.28(-0.87,0.41) | -0.25(-0.68,0.56) |
| Length at discharge | 1 | -0.52(-0.77,0.03) | -0.55(-1.38,0.49) | -0.33(-0.69,0.41) |
| Weight at discharge | 1 | -46.9(-89.0,-3.32) | -9.92(-89.5,69.0) | 16.7(-39.1,70.3) |

Abbreviations: ANS, Antenatal corticosteroids; NRDS, neonatal respiratory distress syndrome; BPD, bronchopulmonary dysplasia; IVH, intraventricular hemorrhage; NEC,necrotizing enterocolitis; ROP, retinopathy of prematurity.

^a^BPD among survivors at 28 days.

^b^ROP among infants

^c^Adjusted for assisted reproductive technology,cesarean section,PROM, birth weight z-scores, multiples, inborn and Apgar ＜7 at 5 min
